# Infant iodine status and associations with maternal iodine nutrition, breast-feeding status and thyroid function

**DOI:** 10.1017/S0007114522001465

**Published:** 2023-03-14

**Authors:** Synnøve Næss, Inger Aakre, Tor A. Strand, Lisbeth Dahl, Marian Kjellevold, Ann-Elin M. Stokland, Bjørn Gunnar Nedrebø, Maria Wik Markhus

**Affiliations:** 1 Seafood, Nutrition and Environmental State, Institute of Marine Research (IMR), Bergen, Norway; 2 Centre for International Health, Department of Global Public Health and Primary Care, University of Bergen, Norway; 3 Department of Research, Innlandet Hospital Trust, Lillehammer, Norway; 4 Department of Endocrinology, Stavanger University Hospital, Stavanger, Norway; 5 Department of Internal Medicine, Haugesund Hospital, Haugesund, Norway; 6 Department of Clinical Science, University of Bergen, Bergen, Norway

**Keywords:** Infants, Urinary iodine concentration, Iodine status, Thyroid function, Breast milk iodine concentration, Breast-feeding

## Abstract

Adequate iodine nutrition during infancy is required for normal thyroid function and, subsequently, brain development. However, data on infant iodine status in the first year of life are scarce. This study aimed to describe infant iodine status and further explore its associations with maternal iodine nutrition, breast-feeding status and thyroid function. In this cohort study, 113 infants were followed up at ages 3, 6 and 11 months in Norway. Infant and maternal urinary iodine concentration (UIC), maternal iodine intake, breast milk iodine concentration (BMIC), breast-feeding status and infant thyroid function tests were measured. The median infant UIC was 82 µg/l at the age of 3 months and below the WHO cut-off of 100 µg/l. Infant UIC was adequate later in infancy (median 110 µg/l at ages 6 and 11 months). Infant UIC was associated positively with maternal UIC (*β* = 0·33, 95 % CI (0·12, 0·54)), maternal iodine intake (*β* = 0·30, 95 % CI (0·18, 0·42)) and BMIC (*β* = 0·46, 95 % CI (0·13, 0·79)). Breastfed infants had lower median UIC compared with formula-fed infants at ages 3 months (76 *v*. 190 µg/l) and 6 months (105 *v*. 315 µg/l). Neither infant UIC nor BMIC were associated with infant thyroid function tests. In conclusion, breastfed infants in Norway are at risk of insufficient iodine intake during the first months of life. Maternal iodine nutrition is important for providing sufficient iodine intake in infants, and awareness of promoting adequate iodine nutrition for lactating women should be prioritised.

Iodine is an essential micronutrient that is required for the synthesis of the thyroid hormones triiodothyronine (T3) and thyroxine (T4)^(1)^. Adequate iodine nutrition is particularly important during the first 1000 d of life (from conception until 2 years of age), as sufficient production of thyroid hormones is required for normal growth and development of the brain^(2)^. The WHO has declared iodine deficiency as the single most important cause of preventable brain damage in fetuses and infants^(3)^. Infants are particularly vulnerable to the consequences of iodine deficiency due to high iodine requirements (based on body weight) and an accelerated turnover of intrathyroidal iodine stores^(4)^.

Adequate iodine intake, and further thyroid hormone production, during the first 4–6 months of life is ensured through breast milk or formula^(5)^. Breast milk iodine concentration (BMIC) is dependent on maternal iodine intake; thus, in exclusively breastfed infants, iodine intake is dependent entirely on maternal iodine intake^(6)^. Iodine deficiency has been reported in lactating and postpartum women in several European countries, including Norway^(7–11)^. Furthermore, estimates suggest that up to 50 % of infants in Europe are at risk of iodine deficiency and, consequently, at risk of not achieving their full cognitive potential^(12)^. However, available research data on iodine status in infants during the first year of life are scarce, and very few studies have additionally measured thyroid function^(13)^. A recent review also emphasised that studies measuring the association between iodine status and thyroid function in infancy in regions with mild iodine deficiency are warranted. In addition, the review highlighted that studies should apply a holistic approach and include both mothers and infants^(14)^.

The main aims of this article were to describe infant iodine status and further explore its associations with maternal iodine nutrition, breast-feeding status and thyroid function.

## Methods

### Study design and participants

The present study is a secondary analysis from the ‘Mommy’s Food’ study (NCT02610959), a two-armed randomised controlled trial. In this study, 137 pregnant women were randomly assigned either to receive Atlantic cod (*Gadus morhua*) twice weekly or to continue with their habitual diet from gestational week 20 to 36^(15,16)^. In this secondary analysis, the children of the participants from both study arms were included in an observational cohort design. The women and their infants were followed up at 3, 6 and 11 months postpartum. Further information about the study design and protocol has been published elsewhere^(15)^. The participants were recruited through the Women’s Clinic at Haukeland University Hospital in Health Region West of Norway from January 2016 to February 2017, and the infants were followed from October 2016 to September 2018.

The study visits at ages 3, 6 and 11 months included collection of biological samples (urine, blood and breast milk) and questionnaires regarding demography, anthropometry, dietary intake and breast-feeding status. Because of loss to follow-up and challenges of sampling infants’ blood, some variables have missing data. Furthermore, not all variables were measured at each time point. Table 1 provides the number of participants and data available at each time point.

**Table 1.** Data and number of participants available at each time point

Measurement	3 months	6 months	11 months
Infant UIC	*n* 108	*n* 90	*n* 91
Infant UIC:Cr	*n* 108	*n* 86	*n* 87
Infant TSH	*n* 47	*n* 53	–
Infant fT3	*n* 47	*n* 52	–
Infant fT4	*n* 47	*n* 52	–
Maternal UIC:Cr	*n* 107	*n* 90	
Maternal iodine intake	*n* 88	*n* 62	*n* 72
BMIC	*n* 99	–	–
Breast-feeding status	*n* 108	*n* 90	*n* 64

UIC, urinary iodine concentration; UIC:Cr, urinary iodine-to-creatinine ratio; TSH, thyroid-stimulating hormone; fT3, free triiodothyronine; fT4, free thyroxine; BMIC, breast milk iodine concentration.

### Infant and maternal urinary iodine concentration and urinary creatinine concentration

A single spot urine sample was collected from each infant at ages 3, 6 and 11 months using a Steriset urine collection pack (REF 310019, Steriset Medical Products). A urine collection pad (21 cm × 7 cm) was placed in the disposable diaper 1 to 2 h before the study visit. The urine collection pads were collected during each study visit and further extracted for urine and transferred into plastic containers. Maternal urine was collected from one spot sample at 3 and 6 months postpartum. Urine samples were stored at −20°C in cryotubes (CryoTubeTM Vials Nunc, Thermo Fischer Scientific) pending analysis. Iodine concentrations in the urine samples were determined through inductively coupled plasma MS. Before analysis, the urine samples were defrosted in a refrigerator, diluted with 1 % tetramethylammonium hydroxide and filtered using a sterile membrane filter with a 0·45-μg pore size and single-use syringe. Samples were analysed with an Agilent 7500 via inductively coupled plasma MS at the Institute of Marine Research (IMR) in Bergen, Norway. Samples were analysed against a urine calibration curve (standard addition curve). Certified reference material was used to check the method’s internal validity: Seronorm Trace Elements Urine (Nycomed Pharma) (iodine content: 84 μg/l (range: 72–96 μg/l) and 304 μg/l (range: 260–348 μg/l)). The method’s measurement uncertainty was assessed based on internal reproducibility and analysis of standard reference material. It was set at 20 % in the entire range (2–297 μg/l).

Urinary creatinine concentration was analysed spectrophotometrically using a MAXMAT PL II multidisciplinary diagnostic platform with a creatinine PAP kit (ERBA Diagnostics France SARL). The urine samples were defrosted at room temperature and centrifuged in an Eppendorf (5810R) centrifuge (15 min, 2000 × g, and 4°C). An aliquot of 200 μl was transferred to the test tube and placed in the MAXMAT carousel for analysis. The method was calibrated using one standard and further controlled with two independent controls.

WHO’s reference value for urinary iodine concentration (UIC) during infancy of 100 µg/l was used to assess adequate iodine status^(17)^. Maternal UIC was presented as the urinary iodine-to-creatinine ratio (UIC:Cr) (µg/g) in statistical models to adjust for individual hydration status^(18)^. UIC:Cr in infants was presented only in supplementary data (online Supplementary Table S1) because infant urinary creatinine concentrations can be highly variable, and no standardised reference values are available^(19)^.

### Maternal iodine intake

Maternal iodine intake was self-reported and estimated from a validated iodine-specific FFQ (I-FFQ) developed specifically for this study^(20)^. The validity of the I-FFQ was assessed in the study population of ‘Mommy’s Food’ at gestational week 18. The estimated iodine intake from the I-FFQ showed good and acceptable agreement with the reference methods, 6-d iodine-specific food diary and UIC from six spot samples, respectively. Reproducibility was assessed by comparing the I-FFQ completed in gestational week 18 with the same I-FFQ completed in gestational week 36 in the control group of the study. There was no difference between the estimated total iodine intake at the two time points, and there was a strong correlation between them indicating that the I-FFQ showed good reproducibility. The I-FFQ was, therefore, considered an adequate tool to estimate iodine intake in the postpartum population. Participants completed the I-FFQ at 3, 6 and 11 months postpartum. The participants were asked to report an estimate of their diet during the past 3 months. The I-FFQ comprised sixty iodine-rich food items (milk and dairy products, fish and other seafood, and eggs) and the use of dietary supplements. Total iodine intake (μg/d) was summarised and estimated from food items and dietary supplements. More information regarding the I-FFQ and calculation of iodine intake has been published elsewhere^(20)^.

### Breast milk iodine concentration

The mothers received six 4·5 ml Nunc® CryoTubes® (Merck KGaA) for the collection of breast milk in the beginning, during and at the end of a chosen feed for 2 d (a total of six samples). The participants kept the breast milk samples in their home freezers, then gave them to the researchers during the study visit at 3 months postpartum. The samples were stored further at −80°C at IMR pending analysis for iodine concentration. The six breast milk samples were analysed as one pooled sample. In each sample, 5 ml of deionised water (> 17 M Ω/cm, Nanopure system, Nanopure, Barnstead) and 1 ml of ultrapure tetramethylammonium hydroxide were added before extraction at 90°C ± 3°C for 3 h. The samples were analysed further for iodine concentrations with inductively coupled plasma MS as described previously.

### Breast-feeding status and infant dietary intake

Information regarding breast-feeding status when the infants were in the age of 3 and 6 months was retrieved through 24-h recall during the study visits. At the age of 11 months, information regarding breast-feeding status was obtained from an electronic questionnaire. Breast-feeding status data were used to create the following categories:

- *Breast-feeding*: No use of formula. Exclusively or predominantly breast-feeding.
- *Mixed milk feeding*: Breast-feeding and use of formula.
- *Formula*: No breast-feeding, only use of formula.

In these categories, no distinction was made between predominantly or exclusively breast-feeding, as the infants’ ages varied between 3 and 11 months. Thus, the categories also included infants receiving complementary foods. For the classification of exclusively breastfed infants, WHO’s definition of *exclusively breast-feeding* was used: ‘breast-feeding with no other food or drink, not even water’^(21)^. Under this definition, prescribed medication, vitamins and minerals are not counted as fluids or foods.

Information regarding the infants’ diet was collected in the online questionnaire at ages 3, 6 and 11 months obtained from the mothers. This included a non-validated FFQ, focusing on eating habits adapted to the infant’s age (3, 6 or 11 months), consisting of questions regarding frequency of iodine-rich sources in the Norwegian diet such as iodine-fortified porridge, cow’s milk, yogurt and lean fish.

### Infant thyroid function tests

Infant thyroid-stimulating hormone (TSH), free T3 (fT3) and free T4 (fT4) were measured in serum samples at ages 3 and 6 months. Venous blood samples for serum preparation were collected in 3·5 ml of BD Vacutainer® SST™ vials II Advanced (Becton, Dickinson and Co.) and set to coagulate for a minimum of 30 min before centrifuging (1000–3000 × g, room temperature, 10 min) within 60 min after venepuncture. If venepuncture was not possible, capillary blood was collected from the infant’s heel or fingertip (according to age or body weight) and placed in a BD Microtainer® Blood Collection Tube (Becton, Dickinson and Co.). A Tenderfoot ITC® heel-incision device (Accriva Diagnostics) was used for heel pricks, and a ACCU-CHEK® Safe-T-Pro Plus lancets (Roche Diagnostics) was used for finger pricks. Post-separation, serum samples were stored at –80°C pending analysis at Fürst Medical Laboratory in Oslo, Norway. The serum samples were stored for a maximum of 3 months before analysis. TSH, fT4 and fT3 were analysed using magnetic separation and detection by chemiluminescence, labelling with acridinium ester, on an Advia Centaur XPT Immunoassay system (Siemens Healthcare Diagnostics). For TSH, fT3 and fT4, the analytical CV were 3·1 %, 3·3 % and 4·6 %, respectively. Reference values for infants from Fürst Medical Laboratory were used (TSH (1–12 months): 1·1–8·2 mµ/l, fT3 (0–9 years): 4·5–8·0 pmol/l, and fT4 (0–12 months): 11·0–23·0 pmol/l)^(22)^. Thyroid dysfunction was defined biochemically by thyroid function tests outside reference ranges^(23)^. Overt hypothyroidism was defined as TSH above and fT4 below reference values. Overt hyperthyroidism was defined as TSH below and fT4 above reference values. Subclinical hypothyroidism and hyperthyroidism were defined as TSH above and below reference values, respectively, and normal fT4 values. Isolated hypothyroxinaemia was defined as normal TSH values and fT4 values below reference values.

### Background variables

Maternal and infant background characteristics were retrieved from electronic questionnaires conducted at recruitment (gestational week 18) and 3, 6 and 11 months postpartum. The questionnaires included information regarding maternal age, education level, pre-pregnancy weight and height, nicotine use (smoking and snuff) and gestational week at birth. Also, the questionnaires included questions regarding infant sex, birth weight, current weight and lenght, and specific infant age when measuring anthropometric variables. Premature birth was defined as birth < gestational week 37. Low birth weight was defined as a birth weight < 2500 g. Infant weight-for-age z-scores at ages 3, 6 and 11 months were calculated using the WHO child growth standard and the WHO Anthro Packages^(24)^. Infant ferritin concentration was analysed in serum samples and collected as described for the aforementioned thyroid function tests. Serum ferritin was analysed by an immunoturbidimetric method using an Advia Chemistry XPT system (Siemens Medical Solutions Diagnostica). The CV for serum ferritin concentration was 2·5 %.

### Ethics

The study was approved by the Regional Committees for Medical and Health Research Ethics West (REK 2015/879) and is registered at ClinicalTrials.gov (NCT02610959). The trial complies with the Declaration of Helsinki, and written informed consent was obtained from the mothers after they were given both written and oral information about the study. The participants were told that they could withdraw from the study at any time without giving any reason.

### Statistics

The number of participants recruited to the study was based on the power calculation for the primary outcome of the study (maternal UIC)^(15)^. Because this article was a secondary analysis of the data, we did not perform a *post hoc* power analysis, because this is not recommended^(25,26)^.

Statistical analyses were performed using Statistical Package for the Social Sciences (SPSS) for Windows, version 27 (IBM Corporation). Variables were tested for normality by visual inspection of Q-Q plots and histograms. Descriptive results were reported as proportions (%) for categorical variables. For continuous variables, means and standard deviations, medians or percentiles were reported as appropriate.

We measured the associations between infant UIC and maternal predictors of iodine status (maternal UIC:Cr, iodine intake and BMIC) and infant thyroid function (TSH, fT3 and fT4) in linear mixed models. All data available from the different time points (3, 6 and 11 months) were included in the models. The associations between the variables in the model at different time points were analysed concurrently, including time point as fixed effect (3, 6 and 11 months) and the individual as a random effect (intercept). Thus, the interdependence in repeated observations from each participant was accounted for. We also estimated the extent to which the independent variables were modified by time points by including interaction terms (time × independent variable) in the model if this affected the model. For each model, restricted maximum likelihood estimation methods were used, and a covariance matrix structure was chosen based on the structure that produced the lowest −2 log-likelihood. The models also were stratified by time points in linear models and presented in Supplementary data.

Potential covariates in the adjusted models were included using ‘Purposeful selection of covariates’, as suggested by Hosmer *et al.* ^(27)^. The following covariates were considered: gestational week at birth; sex, weight-for-age z-score; infant ferritin concentration; maternal pre-pregnancy BMI; maternal age and maternal current nicotine use. All potential covariates were assessed in univariate models with the outcome variable and further included if the *P*-value was < 0·25. Variables that were not significant at the traditional *P* < 0·05 level were excluded stepwise if the coefficient for the independent variable did not change considerably (> 20 %). The following covariates (adjustment variables) were included in the models: infant UIC: pre-pregnancy BMI; infant TSH: maternal pre-pregnancy BMI, maternal current nicotine use, infant ferritin concentration; infant fT3: infant sex, gestational week at birth, pre-pregnancy BMI; and infant fT4: gestational week at birth.

## Results

### Characteristics of study participants

A total of 113 infants were followed up in the study at ages 3, 6 and 11 months. Supplementary Fig. S1 provides the flow chart of the study population in this study. Table 2 shows maternal and infant characteristics of the study population. Mean maternal age at recruitment was 29·3 years, and mean gestational week at birth was 40, with 5 % born < gestational week 37. Mean (sd) birth weight was 3491 (536) g, and 3 % had low birth weight (< 2500 g). At 3 months postpartum, 80 % of the infants were breastfed (where of all were exclusively breastfed), while 15 % were mixed milk-fed and 5 % formula-fed. The proportion of infants who were only breastfed decreased to 78 % at 6 months and 58 % at 11 months (where of 3 % and 0 % were exclusively breastfed, respectively). Supplementary Table S2 provides frequency of intake of iodine-rich foods among the infants at different ages.

**Table 2.** Characteristics of mothers and infants in the Mommy’s Food study (Numbers and percentages; mean values and standard deviations)

		Value			
Characteristic	N	n	%		
**Maternal** *					
Age (years)	113				
Mean	29·6				
sd	3·7				
Pre-pregnancy BMI (kg/m^2^)	110				
Median	22·2				
IQR	20·6–24·3				
Education level, *n* (%)	111				
Elementary school		1	0·9		
High school		12	11		
≤ 4 years university/college		30	27		
> 4 years university/college		68	61		
					
**Infant**	*n*				
Age at study visit (months)	108†		3 months	6 months	11 months
Mean			3·3	6·3	11·5
sd			0·3	0·4	0·4
Sex, *n* (%) girls	112	56	50		
Gestational week at birth					
Mean	113		40		
sd			1·9		
Premature (< GW 37)					
*n*			5		
(%)			4		
Birth weight	92				
Mean			3513		
sd			495		
Low birth weight (< 2500 g)					
*n*			2		
(%)			2		
Weight-for-age z-score	102‡		3 months	6 months	11 months
Mean			0·20	0·59	0·34
sd			0·95	0·98	0·95
< 2 sd (underweight)				0	0
*n*			1		
(%)			1		
Breast-feeding status§	108||		3 months	6 months	11 months
Breastfed (%)			80	78	58
Mixed milk-fed (%)			15	18	14
Formula-fed (%)			5	4	28
Age (weeks) at the introduction of complementary foods	77				
Mean			20·2		
sd			4·1		

IQR, interquartile range; GW, gestational week.

* Maternal characteristics retrieved from an electronic questionnaire completed at gestational week 18.

† Available data on: 3 months, *n* 108; 6 months, *n* 90 and 11 months, *n* 91.

‡ Available data on: 3 months, *n* 102; 6 months, *n* 79 and 11 months, *n* 60.

§ The categories ‘breastfed’ (no use of formula, exclusively or predominantly breast-feeding), ‘mixed milk feeding’ (breastfed and formula-fed) and ‘formula-fed’ (no breast-feeding, only use of formula).

|| Available data on: 3 months, *n* 108; 6 months, *n* 90 and 11 months, *n* 64.

### Infant urinary iodine concentration, breast milk iodine concentration and infant thyroid function

Table 3 provides the infant UIC, BMIC and infant thyroid function tests (TSH, fT3 and fT4) at ages 3, 6 and 11 months. The median UIC was below the recommended WHO cut-off (100 µg/l) at the age of 3 months, with a median UIC of 82 µg/l, indicating insufficient iodine status at group level. At ages 6 and 11 months, the median UIC was 110 µg/l, indicating adequate iodine status at group level. Median (IQR) BMIC was 77 (55–130) µg/l at 3 months postpartum.

**Table 3.** Infant UIC, BMIC and infant thyroid function tests (TSH, fT3 and fT4) (Numbers and percentage; mean values and standard deviations)

	3 months		6 months		11 months	
Variable	*n*	Value	*n*	Value	*n*	Value
**UIC** (µg/l)	108		90		91	
Median		82		110		110
p25–p75		54–140		78–190		77–170
Mean		107		147		141
sd		77		102		104
< 100 µg/l (%)		61%		41%		37%
< 50 µg/l (%)		19%		7%		9%
						
**BMIC** (µg/l)	99			–		–
Median		77				
p25–p75		55–130				
Mean		91				
sd		48				
< 100 µg/l (%)		64%				
< 50 µg/l (%)		13%				
						
**TSH** (mIU/l)	47		53			
Mean		2·4		1·9		
sd		1·2		0·87		
p10–p90		1·1–4·6		0·92–3·4		
min–max		0·80–5·5		0·78–3·9		
< ref. range* (1.1)						
*n*		3		7		
%		6		13		
> ref· range* (8.2)						
*n*		0		0		
%		0		0		
						
**fT3** (pmol/l)	47		52			–
Mean		6·8		6·7		
sd		0·74		0·64		
p10–p90		5·9–7·8		5·9–7·5		
min–max		5·2–8·5		5·2–8·3		
< ref. range* (4.5)						
*n*		0		0		
%		0		0		
> ref. range* (8.0)						
*n*		3		2		
%		6		4		
						
**fT4** (pmol/l)	47		52			–
Mean		17·7		17·6		
sd		1·8		1·6		
p10–p90		15·6–20·0		15·2–20·0		
min–max		13·0–21·0		14·5–21·6		
< ref. range* (11.0)						
*n*		0		0		
%		0		0		
> ref. range* (23.0)						
*n*		0		0		
%		0		0		

UIC, urinary iodine concentration; BMIC, breast milk iodine concentration; TSH, thyroid-stimulating hormone; fT3, free triiodothyronine; fT4, free thyroxine.

* Reference ranges derived from laboratory^(22)^. TSH (1–12 months): 1·1–8·2 mµ/l; fT3 (0–9 years): 4·5–8·0 pmol/l; fT4 (0–12 months): 11·0–23·0 pmol/l.

Few infants were outside the reference ranges for thyroid function tests (TSH, fT3 and fT4) (Table 3). None of the infants had fT4 concentrations outside reference ranges or TSH concentrations above reference ranges at either of the time points. Furthermore, none of the infants were defined with biochemically assessed thyroid dysfunction in the categories of overt hypothyroidism, overt hyperthyroidism, subclinical hypothyroidism or isolated hypothyroxinaemia. At ages 3 and 6 months, respectively, 6 % and 13 % of the infants had TSH concentrations below the reference range which can be defined as biochemically assessed subclinical hyperthyroidism.

### Infant urinary iodine concentration and associations with maternal urinary iodine-to-creatinine ratio, iodine intake and breast milk iodine concentration

Table 4 provides the associations between infant UIC and maternal UIC:Cr and iodine intake and BMIC in repeated measurement analyses. Infant UIC was associated positively (adjusted coefficient (95 % CI)) with maternal UIC:Cr (0·33 (0·12, 0·54), *P* = 0·002), maternal iodine intake (0·30 (0·18, 0·42), *P* < 0·001) and BMIC (0·36 (0·064, 0·66), *P* = 0·018). The same associations were found when using infant UIC:Cr in the models (online Supplementary Table S3). The time interaction (time × independent variable) did not affect either of the models, but we also included the models stratified by time points (online Supplementary Table S4A–C and S5A–C). The associations between infant UIC and maternal UIC:Cr and iodine intake were strongest at the age of 3 months.

**Table 4.** Associations between infant UIC and maternal indicators of iodine nutrition in Linear Mixed Models (Coefficient and 95 % confidence intervals)

	Infant UIC					
	Unadjusted			Adjusted§		
Independent variables	Coefficient	95% CI	*P*	Coefficient	95% CI	*P*
Maternal UIC:Cr*	0·31	0·10, 0·51	0·003	0·33	0·12, 0·54	0·002
Maternal iodine intake†	0·29	0·17, 0·41	< 0·001	0·30	0·18, 0·42	< 0·001
BMIC‡	0·35	0·06, 0·65	0·018	0·35	0·05, 0·66	0·022

UIC, urinary iodine concentration; UIC:Cr, urinary iodine-to-creatinine ratio; BMIC, breast milk iodine concentration.

* The model includes infant UIC and maternal UIC/Cr at two time points (ages 3 and 6 months).

† The model includes infant UIC and maternal estimated total iodine intake (foods and supplements) at three time points (ages 3, 6 and 11 months).

‡ The model includes infant UIC and BMIC at one time point (age 3 months).

§ Covariates in adjusted model: maternal pre-pregnancy BMI.

### Infant urinary iodine concentration and associations with breast-feeding status

**Fig. 1.**
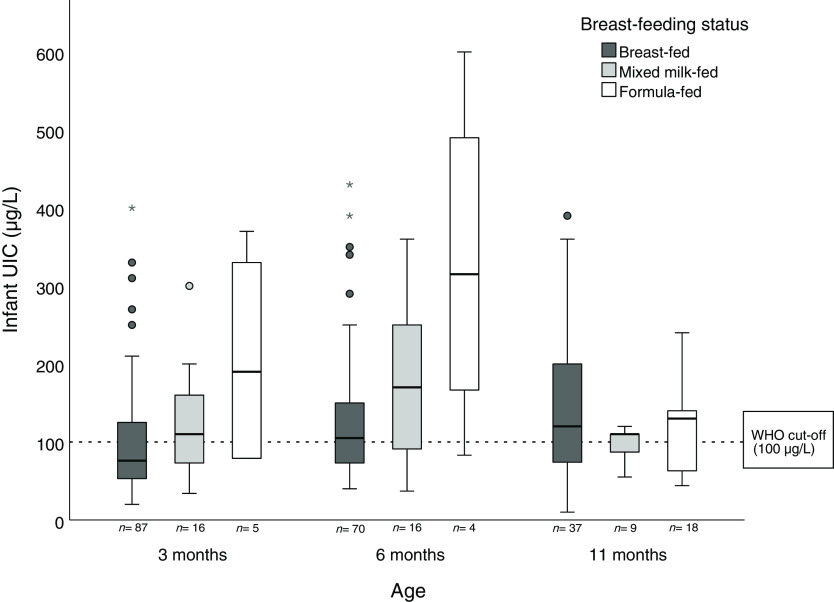
Box plot of infant urinary iodine concentration (UIC) at ages 3, 6 and 11 months by breast-feeding status categories. The boxes indicate the upper (75th percentile) and lower (25th percentile) quartiles with the thick black line as the median (50th percentile). The T-bars indicate 1·5 × length of the box (interquartile range). The filled circles are outliers, defined as a value > 1·5 length of the box. The asterisks are extreme outliers, defined as a value > 3·0 length of the box.

Infant UIC at ages 3, 6 and 11 months categories of breast-feeding status are shown in Fig. 1. At ages 3 and 6 months, breastfed infants had a lower median UIC (76 and 105 µg/l, respectively) compared with formula-fed infants (190 and 315 µg/l, respectively). At the age of 11 months, no apparent differences were found between breast-feeding status categories. Similar patterns were also found between infant UIC:Cr and breast-feeding status (online Supplementary Fig. S2).

### Infant urinary iodine concentration and breast milk iodine concentration and associations with infant thyroid function

Associations between infant UIC and BMIC with TSH, fT3 and fT4 are provided in Table 5. No associations were found between either of the variables.

**Table 5.** Associations between infant UIC and BMIC and infant TSH, fT3 and fT4 concentrations (Coefficient and 95 % confidence intervals)

	Dependent variables					
	Unadjusted			Adjusted‡		
	Coefficient	95% CI	*P*	Coefficient	95% CI	*P*
Independent variables	Infant TSH					
Infant UIC*	–0·06	–0·27, 0·16	0·599	0·13	–0·22, 0·48	0·459
BMIC†	–0·22	–0·92, 0·47	0·515	–0·46	–1·15, 0·22	0·178
	Infant fT3					
Infant UIC*	0·01	–0·04, 0·23	0·149	0·06	–0·06, 0·19	0·324
BMIC†	–0·13	–0·55, 0·30	0·555	–0·07	–0·45, 0·32	0·414
	Infant fT4					
Infant UIC*	0·016	–0·22, 0·53	0·408	0·13	–0·24, 0·51	0·471
BMIC†	0·11	–0·90, 1·12	0·822	0·13	–0·87, 1·12	0·801

UIC, urinary iodine concentration; BMIC, breast milk iodine concentration; TSH, thyroid-stimulating hormone; fT3, free triiodothyronine; fT4, free thyroxine.

* The model includes two time points (infants, 3 and 6 months of age) in linear mixed models. The coefficient is given in per 100 µg/l.

† The model includes one time point (infant, 3 months of age) in linear models. The coefficient is given in per 100 µg/l.

‡ Covariates in adjusted model: TSH: maternal pre-pregnancy BMI, maternal current nicotine use, infant ferritin concentration; fT3: infant sex, gestational week born, maternal pre-pregnancy BMI; fT4: gestational week born.

## Discussion

This is the first study to present data on both iodine status and thyroid function in Norwegian infants during the first year of life. We found that the infants had insufficient iodine status at the age of 3 months, as indicated by a median UIC of 82 µg/l. Infant UIC increased during the first year of life, with median UIC of 110 µg/l at ages 6 and 11 months. Breastfed infants had substantially lower UIC compared with formula-fed infants at ages 3 and 6 months. Furthermore, infant UIC was associated with maternal markers of iodine nutrition (UIC:Cr, iodine intake and BMIC). However, no associations were found between infant UIC and thyroid function, and the prevalence of infant thyroid dysfunction was low.

Using the WHO criteria for sufficient iodine status of 100 µg/l in children aged < 2 years, the infants in this study had insufficient iodine status at the age of 3 months, but adequate status at ages 6 and 11 months. The WHO criteria of optimal iodine status in infants has been debated^(6,13,28)^, and a dose–response cross-over iodine balance study in Swiss euthyroid infants suggested 125 µg/l as being an appropriate new cut-off for the infant median UIC^(13)^. Using the latter cut-off, the median UIC for infants in this study was insufficient at all time points. Nevertheless, our results indicate that infants are most vulnerable to iodine deficiency during the first months of life.

Breastfed infants had lower median UIC compared with formula-fed infants at ages 3 months (76 *v*. 190 µg/l) and 6 months (105 *v*. 315 µg/l). At the age of 3 months, 80 % of the infants were exclusively breastfed and their iodine intake accordingly relied completely on maternal iodine intake. In this study, median BMIC at 3 months postpartum was 77 µg/l, which is considerably lower than the iodine concentration in formula on the European market (about 130 µg/l)^(29)^. Assuming a breast milk intake of 0·75 l/d at the age of 3 months^(30)^, a BMIC of 77 µg/l, would correspond to an iodine intake of 58 µg/d in fully breastfed infants in the present study. This is much lower than the WHO recommendation of 90 µg/d^(3)^, which corresponds to our results, indicating an inadequate iodine status in exclusively breastfed infants. Lower iodine status in breastfed infants compared with formula-fed infants has also been reported in other Norwegian studies^(31,32)^ in addition to international studies^(33–35)^. In the current study, infant UIC was associated with all markers of maternal iodine nutrition, including BMIC, UIC:Cr and iodine intake. Consequently, this confirms that maternal iodine nutrition is the most important factor for providing sufficient iodine status in breastfed infants. However, the associations were strongest at the age of 3 months, indicating that maternal iodine nutrition’s significance decreases during the first year of life when the infant is introduced to other iodine sources than breast milk. This was also supported by the increase of iodine-rich complementary foods and formula during the first year of life in this study. In this study, the mean age of introducing complementary foods to infants was 5 months, which corresponds to other infants in Norway^(36)^. Likewise, the intake of iodine-rich food sources, such as iodine-fortified porridge, cow’s milk, yogurt and lean fish, increased from age 6 to 11 months (online Supplementary Table S2).

Previously, we reported insufficient maternal iodine status in this group at 3 and 6 months postpartum (median UIC of 74 and 84 µg/l, respectively)^(37)^. In Norway, iodine supplementation recently has been recommended (from 2018) for lactating women if intake of the most important iodine sources (dairy products and lean fish) is low^(38)^. The WHO also recommends iodine supplementation for lactating women in regions with insufficient access to iodised salt and where few dietary iodine sources are available^(3)^. In the present study, at 3 and 6 months postpartum, only 30 % and 17 % of the mothers used an iodine-containing supplement, respectively^(37)^. Considering that exclusive breast-feeding is recommended during the first 6 months^(39)^, achieving adequate iodine status for lactating women is crucial in providing sufficient iodine intake for infants.

A recent study from Norway found that infants (median age 5 months, range 0–12 months) had adequate iodine status with a median UIC of 146 µg/l^(31)^, which is substantially higher that in our study. Higher iodine status also have been reported in Norwegian toddlers at the age of 18 months (median UIC 129 µg/l)^(40)^. However, a higher mean age, lower breast-feeding rates and higher iodine intake from complementary foods in the two studies may explain the differences compared with our results. Unlike our results, several studies have not found an association between infant UIC and breast-feeding status^(41–44)^. However, compared with our study, these studies reported adequate infant UIC and higher BMIC. Thus, in lactating women with sufficient iodine status, there may be no differences between iodine concentration in breast milk and formula. Consequently, both breastfed and formula-fed infants receive adequate iodine status. It should also be noted that the difference in infant iodine status between breast-feeding categories in our study (breastfed, mixed milk-fed and formula-fed) was negligible and at 11 months. At this age, however, the intake of complementary iodine sources was higher (online Supplementary Table S2) and the explained variance in infant UIC by breast-feeding status was also expected to decrease.

Generally, infancy is the period in life with the highest vulnerability of developing thyroid disorders due to iodine deficiency^(45)^. Even though infant iodine deficiency was present in the first months of life, the prevalence of thyroid dysfunction was low and none of the infants had thyroid functions tests indicating subclinical or overt hypothyroidism. Furthermore, we did not find any association between infant UIC or BMIC with either TSH, fT3 or fT4. However, thyroid function data were available from only half the infants, providing low power to detect any potential associations. Also, the infants in this study did not suffer from severe iodine deficiency, and adequate thyroid hormone production is typically maintained under mild iodine deficiency^(46)^. Nevertheless, a randomised controlled trial with iodine supplements in mildly iodine-deficient school age children demonstrated improved iodine status and cognitive function without thyroid hormone concentrations being affected^(47)^. Thus, the insufficient iodine status of the infants in this study may have consequences for infant neurodevelopment, even though we did not observe any association with thyroid function. However, the data assessing the risk of mild iodine deficiency on thyroid function during infancy is sparse, and further studies are needed to assess the vulnerability of iodine deficiency in this group^(14)^.

### Strengths and limitations

This study has several strengths. Infant UIC and thyroid function were measured at several time points during the first year of life, which further improves the measurements’ reliability. Furthermore, several markers of maternal iodine nutrition (UIC:Cr, BMIC and iodine intake captured from a validated I-FFQ) were measured, providing a comprehensive measurement of the infants’ iodine nutrition and possible explanatory variables. However, this study also has some limitations that should be acknowledged. The study comprised a limited sample size from one region of Norway, so the results may not be generalisable directly to all infants in Norway. Furthermore, the significant amount of missing data on infant thyroid function is an important shortcoming. Also, the categorisation of breast-feeding status yielded few participants in the formula-fed and mixed milk feeding groups; thus, the observed differences should be interpreted with caution. Finally, spot UIC can be used only to evaluate iodine status at a group level; therefore, we could not determine what percentage of the infants that truly were iodine-deficient.

### Conclusion

Infant iodine status was insufficient at the age of 3 months, but adequate at ages 6 and 11 months. Furthermore, breastfed infants had lower iodine status compared with formula-fed infants. This indicates that weaned and formula-fed infants are less vulnerable to iodine deficiency, considering that iodine intake is introduced through complementary foods and formula. Lactating women in Norway have inadequate iodine intake; therefore, the iodine content of breast milk may be insufficient to meet fully breastfed infants’ dietary iodine requirements. Although no consequences on thyroid function were found, maternal iodine nutrition is important for providing sufficient iodine intake in infants. Awareness of promoting adequate iodine nutrition for lactating women should be prioritised to secure sufficient iodine intake for mothers and, subsequently, their infants.

## Supplementary material

For supplementary material accompanying this paper visit https://doi.org/10.1017/S0007114522001465. click here to view supplementary material
