# Supplementary material for: Infant iodine status and associations with maternal iodine nutrition, breast-feeding status and thyroid function
Source: Br J Nutr. 2022 May 10;129(5):854–63. doi: 10.1017/S0007114522001465 (PMC9975782; doi:10.1017/S0007114522001465)
Supplement: Supplementary file 1 [file S0007114522001465sup001.docx]

## Supplementary Data

**Supplementary Figure S1:** Flow chart of the study population and data available at each time-point.

**Supplementary Figure S2**: Box plot of infant urinary iodine concentration: creatinine concentration (UIC:Cr) (µg/g) at age 3, 6 and 11 months by categories of breastfeeding status.

**Supplementary Table S1:** Infant creatinine and UIC:Cr at age 3, 6 and 11 months

**Supplementary Table S2:** Frequency of intake (times/week) of iodine-rich foods among Norwegian infants

**Supplementary Table S3:** Associations between infant UIC:Cr with maternal predictors of iodine nutrition

**Supplementary Table S4A:** Associations between infant UIC with maternal indicators of iodine nutrition at age 3 months

**Supplementary Table S4B:** Associations between infant UIC with maternal indicators of iodine nutrition at age 6 months

**Supplementary Table S4C:** Associations between infant UIC with maternal indicators of iodine nutrition at age 11 months

**Supplementary Table S5A:** Associations between infant UIC:Cr with maternal indicators of iodine nutrition at age 3 months

**Supplementary Table S5B:** Associations between infant UIC:Cr with maternal indicators of iodine nutrition at age 6 months

**Supplementary Table S5C:** Associations between infant UIC:Cr with maternal indicators of iodine nutrition at age 11 months


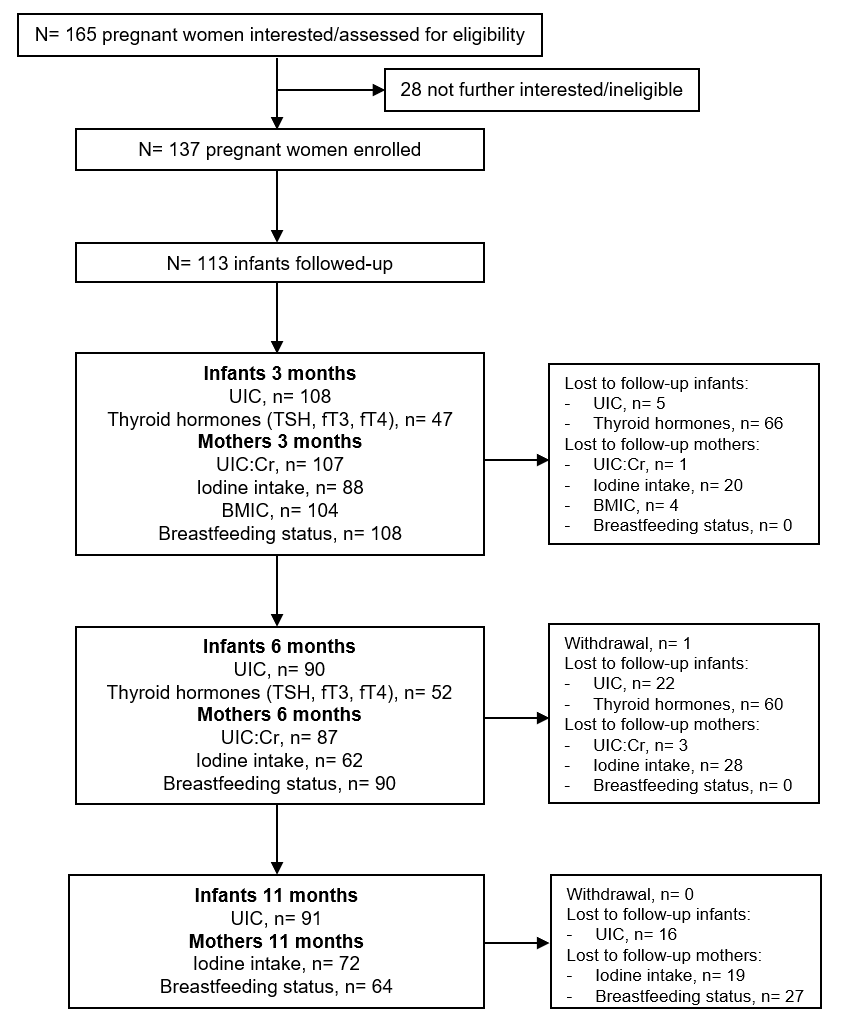


**Figure S1** Flow chart of the study population and data available at each time-point.

BMIC, breast-milk iodine concentration; fT3, free triiodothyronine; fT4, free thyroxine; TSH, thyroid-stimulating hormone; UIC, urinary iodine concentration. UIC:Cr, urinary iodine to creatinine ratio


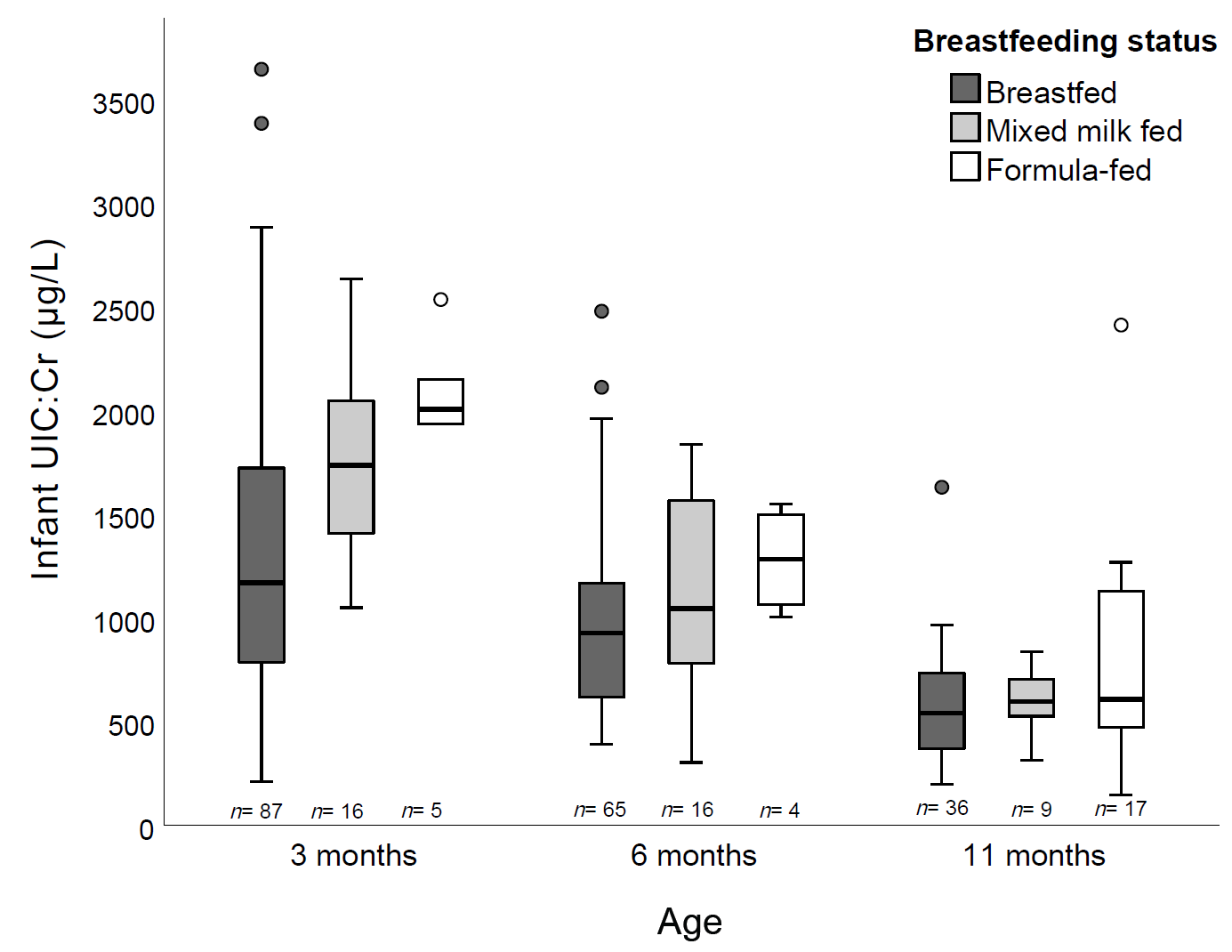
**Figure S2** Box plot of infant urinary iodine concentration: creatinine concentration (UIC:Cr) (µg/g) at age 3, 6 and 11 months by categories of breastfeeding status.

Boxes indicates the upper (75th percentile) and lower (25th percentile) quartile with the thick black line giving the median (50th percentile). The T-bars indicate 1.5 × length of the box (inter quartile range). The filled circles are outliers defined as a value > 1.5 length of the box. The asterisks are extreme outliers defined as a value > 3.0 length of the box.

**Table S1** Infant creatinine and UIC:Cr at age 3, 6 and 11 months

|  | N | **3 M** | N | **6 M** | N | **11 M** |
| --- | --- | --- | --- | --- | --- | --- |
| **Creatinine (g/L)**  Mean (SD)  Median  (p25-p75) | 108 | 0.077 (0.046)  0.066  (0.05, 0.09) | 86 | 0.15 (0.093)  0.12  (0.08, 0.18) | 87 | 0.24 (0.17)  0.18  (0.11, 0.32) |
| **UIC:Creatinine ratio (µg/g)**  Mean (SD)  Median  (p25-p75) | 108 | 1524 (1015)  1305  (852-1958) | 86 | 1053 (617)  964  (641-1238) | 87 | 655 (372)  580  (453-795) |

UIC, urinary iodine concentration

**Table S2** Frequency of intake (times/week) of iodine-rich foods among Norwegian infants ^a^

|  | **Age** | |
| --- | --- | --- |
|  | 6 months (*n*= 77) | 11 months (*n*= 82) |
| **Iodine-rich foods** | n (%) | n (%) |
| Porridge (industry manufactured)  Never/rarely  1 time/week  2-3 times/week  4-6 times/week  Daily | 22 (28)  4 (5)  4 (5)  12 (16)  35 (46) | 12 (15)  5 (6)  4 (5)  9 (11)  52 (63) |
| Porridge (homemade)  Never/rarely  1 time/week  2-3 times/week  4-6 times/week  Daily | 56 (73)  2 (3)  6 (8)  4 (5)  9 (12) | 49 (60)  5 (6)  4 (5)  9 (11)  11 (13) |
| Cow milk  Never/rarely  1 time/week  2-3 times/week  4-6 times/week  Daily | 77 (100)  -  -  -  - | 67 (82)  5 (6)  3 (4)  4 (5)  3 (4) |
| Yoghurt  Never/rarely  1 time/week  2-3 times/week  4-6 times/week  Daily | 76 (99)  -  1 (1)  -  - | 31 (38)  14 (17)  20 (24)  10 (12)  7 (8) |
| Lean fish, dinner  Never/rarely  1 time/week  2-3 times/week  4-6 times/week  Daily | 67 (87)  8 (10)  2 (3)  -  - | 18 (22)  54 (66)  8 (10)  2 (2)  - |
| Fish products (fish cakes, fish au gratin, fish fingers, fish pudding)  Never/rarely  1 time/week  2-3 times/week  4-6 times/week  Daily | 77 (100)  -  -  -  - | 27 (33)  38 (47)  14 (17)  2 (3)  - |

^a^ Frequency of intake at infants ages 6 and 11 months reported by the mothers in an electronic food frequency questionnaire (FFQ). At age 3 months, ‘never/rarely’ was reported among all participants in all food categories.

**Table S3** Associations between infant UIC:Cr and maternal predictors of iodine nutrition

|  | **Infant UIC:Cr** | | | |
| --- | --- | --- | --- | --- |
|  | Unadjusted | | Adjusted ^d^ | |
| **Independent variables** | Coefficient  (95% CI) | *P* | Coefficient  (95% CI) | *P* |
| Maternal UIC:Cr ^a^ | 5.5  (3.7, 7.3) | <0.001 | 5.9  (4.0, 7.8) | <0.001 |
| Maternal iodine intake ^b^ | 1.9  (1.0, 2.9) | <0.001 | 1.9  (1.0, 2.9) | <0.001 |
| BMIC ^c^ | 5.2  (1.3, 9.2) | 0.011 | 5.6  (1.5, 9.8) | 0.009 |

Linear Mixed Models.

BMIC, breast milk iodine concentration; UIC, urinary iodine concentration; UIC:Cr, iodine-to-creatinine ratio

^a^ Model includes infant UIC:Cr and maternal UIC:Cr at two time points (infant 3 and 6 months of age).

^b^ Model includes infant UIC:Cr and maternal estimated total iodine intake (foods and supplements) at three time points (infant 3, 6 and 11 months of age).

^c^ Model includes infant UIC:Cr and BMIC at one time-point (infant 3 months of age).

^d^ Covariates in adjusted model: Maternal pre-pregnancy BMI

**Table** **S3A** Associations between infant UIC and maternal indicators of iodine nutrition at age 3 months

|  | **Infant UIC 3 months** | | | |
| --- | --- | --- | --- | --- |
|  | Unadjusted | | Adjusted ^a^ | |
| **Independent variables** | Coefficient  (95% CI) | *P* | Coefficient  (95% CI) | *P* |
| Maternal UIC:Cr | 0.36  (0.14, 0.57) | 0.002 | 0.37  (0.15, 0.59) | 0.001 |
| Maternal iodine intake | 0.28  (0.12, 0.43) | 0.001 | 0.30  (0.15, 0.45) | <0.001 |
| BMIC | 0.36  (0.07, 0.65) | 0.016 | 0.36  (0.06, 0.66) | 0.018 |

Linear Models.

BMIC, breast milk iodine concentration; UIC, urinary iodine concentration; UIC:Cr, iodine-to-creatinine ratio

^a^ Covariates in adjusted model: Maternal pre-pregnancy BMI

**Table S3B** Associations between infant UIC and maternal indicators of iodine nutrition at age 6 months

|  | **Infant UIC 6 months** | | | |
| --- | --- | --- | --- | --- |
|  | Unadjusted | | Adjusted ^a^ | |
| **Independent variables** | Coefficient  (95% CI) | *P* | Coefficient  (95% CI) | *P* |
| Maternal UIC:Cr | 0.25  (-0.33, 0.84) | 0.391 | 0.29  (-0.30, 0.88) | 0.325 |
| Maternal iodine intake | 0.38  (0.12, 0.64) | 0.005 | 0.40  (0.14, 0.65) | 0.003 |

Linear Models.

UIC, urinary iodine concentration; UIC:Cr, iodine-to-creatinine ratio

^a^ Covariates in adjusted model: Maternal pre-pregnancy BMI

**Table S3C** Associations between infant UIC and maternal indicators of iodine nutrition at age 11 months

|  | **Infant UIC 11 months** | | | |
| --- | --- | --- | --- | --- |
|  | Unadjusted | | Adjusted ^a^ | |
| **Independent variable** | Coefficient  (95% CI) | *P* | Coefficient  (95% CI) | *P* |
| Maternal iodine intake | 0.24  (-0.01, 0.49) | 0.058 | 0.23  (-0.02, 0.49) | 0.075 |

Linear Models.

^a^ Covariates in adjusted model: Maternal pre-pregnancy BMI

**Table S4A** Associations between infant UIC:Cr with maternal indicators of iodine nutrition at age 3 months

|  | **Infant UIC** | | | |
| --- | --- | --- | --- | --- |
|  | Unadjusted | | Adjusted ^a^ | |
| **Independent variables** | Coefficient  (95% CI) | *P* | Coefficient  (95% CI) | *P* |
| Maternal UIC:Cr | 7.4  (4.8, 10.1) | <0.001 | 8.2  (5.4, 10.9) | <0.001 |
| Maternal iodine intake | 4.9  (2.9, 6.8) | <0.001 | 5.3  (3.3, 7.3) | <0.001 |
| BMIC | 5.2  (1.3, 9.2) | 0.011 | 5.6  (1.5, 9.8) | 0.009 |

Linear Models.

BMIC, breast milk iodine concentration; UIC, urinary iodine concentration; UIC:Cr, iodine-to-creatinine ratio

^a^ Covariates in adjusted model: Maternal pre-pregnancy BMI

**Table S4B** Associations between infant UIC:Cr with maternal indicators of iodine nutrition at age 6 months

|  | **Infant UIC** | | | |
| --- | --- | --- | --- | --- |
|  | Unadjusted | | Adjusted ^a^ | |
| **Independent variables** | Coefficient  (95% CI) | *P* | Coefficient  (95% CI) | *P* |
| Maternal UIC:Cr | 4.3  (0.85, 7.9) | 0.016 | 4.4  (0.83, 8.0) | 0.016 |
| Maternal iodine intake | 3.0  (1.4, 4.7) | 0.001 | 3.1  (1.4, 4.8) | 0.001 |

Linear Models.

UIC, urinary iodine concentration; UIC:Cr, iodine-to-creatinine ratio

^a^ Covariates in adjusted model: Maternal pre-pregnancy BMI

**Table S4C** Associations between infant UIC:Cr with maternal indicators of iodine nutrition at age 11 months

|  | **Infant UIC** | | | |
| --- | --- | --- | --- | --- |
|  | Unadjusted | | Adjusted ^a^ | |
| **Independent variable** | Coefficient  (95% CI) | *P* | Coefficient  (95% CI) | *P* |
| Maternal iodine intake | 0.85  (-0.15, 1.85) | 0.095 | 0.77  (-0.27, 1.8) | 0.146 |

Linear Models.

^a^ Covariates in adjusted model: Maternal pre-pregnancy BMI
